# Clinical Features of Type B Insulin Resistance in Japanese Patients: Case Report and Survey-Based Case Series Study

**DOI:** 10.1155/2020/4359787

**Published:** 2020-04-04

**Authors:** Yusuke Hirota, Hirotsugu Suwanai, Toshimasa Yamauchi, Takashi Kadowaki

**Affiliations:** ^1^Department of Diabetes and Metabolic Diseases, Graduate School of Medicine, The University of Tokyo, Tokyo 113-8655, Japan; ^2^Department of Diabetes, Metabolism and Endocrinology, Tokyo Medical University, Tokyo 160-0023, Japan; ^3^Department of Prevention of Diabetes and Lifestyle-Related Diseases, Graduate School of Medicine, The University of Tokyo, Tokyo 113-8655, Japan; ^4^Department of Metabolism and Nutrition, Mizonokuchi Hospital, Teikyo University, Kanagawa 213-8507, Japan

## Abstract

Type B insulin resistance (TBIR) is an extremely rare disease characterized by marked hyperglycemia and insulin resistance and often coexists with autoimmune diseases. The characteristics, symptoms, blood glucose patterns, comorbidities, and treatments of TBIR all vary and are not defined. In this study, we described a case of TBIR that developed 6 months after DPP-4 inhibitor administration and immediately after the patient caught a cold. Treatment using prednisolone and insulin-like growth factor-1 was effective. We also conducted an observational survey-based case series study in a Japanese cohort comprising 21 cases. The average age of onset of TBIR was 62.3 ± 14.8 (17–84) years, and 61.9% of subjects were male. The majority of patients (90.4%) were 50 years old and over. During the study period, there was a high percentage (85.7%) of episodes of hypoglycemia, which was the trigger for diagnosis in more than 50% of cases. Glycemic patterns included 7 cases of hyperglycemia (33.3%), 10 cases of hypoglycemia (47.6%), and 4 cases of both hyperglycemia and hypoglycemia (19.1%). In the hypoglycemic group, 90.0% of patients were male. Furthermore, 71.4% of cases were antinuclear antibody positive, and 81.0% of cases were complicated with autoimmune disease. Systemic lupus erythematosus (38.1%) and Sjögren's syndrome (23.8%) were relatively common as coexisting autoimmune diseases. Treatment was based on prednisolone use, which was used in 88.9% of patients. On the other hand, the effect of IGF-1 was limited. Overall, the prognosis of TBIR was good.

## 1. Introduction

Type B insulin resistance (TBIR) is an autoimmune disease caused by the presence of immunoglobulin G (IgG) polyclonal antibodies, which competitively inhibit the binding of insulin to the insulin receptor [1, 2]. This condition is extremely rare, and only 67 cases have been reviewed in the literature as of June 2014 [1]. TBIR is characterized by marked hyperglycemia and insulin resistance and is refractory to the administration of large amounts of insulin. TBIR and autoimmune diseases, such as systemic lupus erythematosus (SLE), often coexist [1]. In cases in which an obvious autoimmune disease cannot be diagnosed, autoimmune abnormalities, such as antinuclear antibody positive, are often detected [3]. The typical findings include acanthosis nigricans and extreme weight loss, but the symptoms vary.

TBIR disease has been reported in Asians, and the characteristic clinical findings in Asian patients, namely, high incidence of hypoglycemia, low incidence of acanthosis nigricans, and low number of required insulin units, have also been reported [4]. There is no standard treatment for TBIR because of the differences in antibody titers, antibody effects, and underlying diseases; therefore, treatment is tailored to each case [5]. The reported examples of treatments include steroids [4], immunosuppressants [6], *Helicobacter pylori* eradication therapy [7], plasma exchange therapy [6], anti-CD20 monoclonal antibodies [8], combination of a steroid-immunosuppressant drug and anti-CD20 monoclonal antibody [5, 9–11], and insulin-like growth factor-1 [6, 12, 13].

We identified a case of TBIR. During the management of this case, we found that there was little information about TBIR in Japanese patients. Therefore, we conducted an observational survey-based case series study in Japanese patients. A study in 1994 summarized 16 Japanese cases of TBIR [14]. A recent report summarized Japanese cases including the patient's age, insulin level, BMI, HbA1c, autoimmune disease, immunomodulation therapy, treatment for diabetes, and presence of hypoglycemia [15]. However, the classification, comparison, and characteristics for each blood glucose pattern (hyperglycemia, hypoglycemia, or both hyperglycemia and hypoglycemia), statistical analysis, prognosis, and specific effects of IGF-1 have not been clarified. Our study is the first to analyze and clarify these. Moreover, we examined the hypothesis that DPP-4 inhibitors and gender differences may affect TBIR development. Considering that this disease has diverse characteristics, pathophysiology, symptoms, blood glucose fluctuations, comorbidities, and treatment methods, a survey-based case series study should be conducted, particularly in a Japanese cohort, because there is inadequate information and analyses from various perspectives about this disease in the Japanese population. The clarification of such information will help the diagnosis and treatment of TBIR and contribute to further studies.

## 2. Materials and Methods

### 2.1. Literature Search

**Figure 1:**
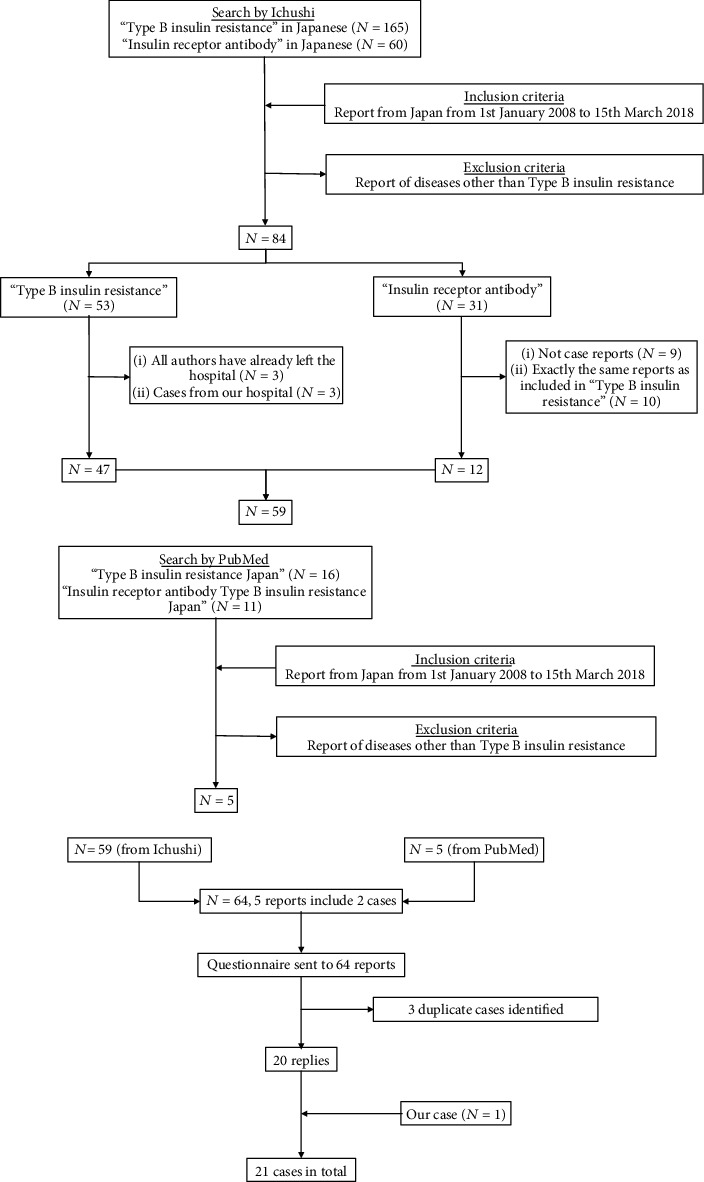
Flow chart showing the selection of study cases.

This survey was an observational survey-based case series study. A survey was conducted by sending a questionnaire to the authors of Japanese cases reported from January 1, 2008, to March 15, 2018.  Figure 1 shows the details of the case search.

Questionnaires were sent to 64 selected reports. The survey focused specifically on blood glucose level and its related values, blood glucose patterns, process leading to diagnosis, treatment, use of dipeptidyl peptidase-4 (DPP-4) inhibitor, outcomes, prognosis, and comorbid diseases. Cases were divided into three groups based on the blood glucose pattern. Each survey item was compared between the three groups.

The study was approved by the ethics committee of the Graduate School of Medicine and the Faculty of Medicine, The University of Tokyo (Credit No. 11958-(1)). All statistical analyses were performed using EZR version 1.40 (Saitama Medical Center, Jichi Medical University, Saitama, Japan).

### 2.2. Case Study

In this study, we describe the successful treatment of a patient with TBIR.

A 68-year-old Japanese man developed marked hyperglycemia and was hospitalized and diagnosed with TBIR. He had been diagnosed with type 2 diabetes at the age of 47 years and was treated with an oral hypoglycemic drug. The administration of sitagliptin (50 mg/day) was initiated at 6 months prior to hospitalization. The patient developed a cold (symptoms included chills and a runny nose) 1 week prior to hospitalization. Self-monitored blood glucose levels on the day before hospitalization were very high; thus, he visited our hospital.

The patient's medical history included meningioma, splenic infarction, colon cancer, and chronic renal failure. However, he had no history of mental illness. His family history was as follows: his father had diabetes, his mother had high blood pressure and chronic renal failure, and his sister had colon cancer. The oral medications included sitagliptin (50 mg/day), glimepiride (0.5 mg/day), irbesartan (50 mg/day), nifedipine (40 mg/day), furosemide (40 mg/day), spironolactone (25 mg/day), aspirin (100 mg/day), famotidine (20 mg/day), allopurinol (50 mg/day), and rosuvastatin (5 mg/day). He had not used insulin injection. His lifestyle history was as follows: he used to smoke 100 cigarettes per day from the age of 20 to 68 years but quit smoking after that. He consumed 750 mL of beer per day from the age of 20 to 65 years. He was unemployed.

Physical examination revealed a waist circumference of 107.0 cm and paralyzed left upper and lower limbs (due to sequela of meningioma surgery). The results of the examination showed a random blood glucose level of 587 mg/dL (32.6 mmol/L), immunoreactive insulin (IRI) level of 1340 *μ*U/mL, and C-peptide (CPR) level of 15.7 ng/mL (5.20 nmol/L) (Because the blood glucose level was very high, IRI and CPR were tested using the same specimen.) (Table 1). The patient was hospitalized the next day to investigate and treat hyperglycemia and hyperinsulinemia.

On the day of hospitalization, the blood tests showed hemoglobin A1c (HbA1c) of 7.2% (55.2 mmol/mol) and glycoalbumin of 28.1% (Table 1). There was divergence between HbA1c and glycoalbumin values, suggesting a sharp increase in blood glucose levels. On the second day of hospitalization, the fasting blood glucose (FBG) levels were 538 mg/dL (29.9 mmol/L), and the fasting CPR levels were 10.3 ng/mL (3.4 nmol/L). It was impossible to measure the fasting IRI because it was of extremely high value and was affected by the influence of insulin antibody (Table 2).

Despite gradually increasing the use of insulin from 10 units/day to 170 units/day in 20 days after hospitalization, blood glucose levels did not decrease sufficiently. Differential diagnosis exhibiting marked insulin resistance is diverse. As a result, the possibility of obesity, chronic kidney disease, and insulin receptor abnormality remained positive in this case; the various physical findings and tests for other conditions were negative. Given that the patient was anti-insulin receptor antibody positive with a binding rate of 71.3%, he was diagnosed with TBIR.

We investigated the presence of comorbid disorders such as collagen diseases, including SLE. Physical findings did not indicate collagen diseases, and various autoantibodies were negative. Considering that antinuclear antibodies were positive only for cytoplasmic type, this did not enable the diagnosis of a specific disease. We also considered the possibility of malignant tumors. There were no significant findings in the chest X-ray examination, abdominal ultrasonography, whole-trunk computed tomography examination, and upper and lower gastrointestinal endoscopy. Endocrine disorders were also negative. *H. pylori* infection was negative.

**Figure 2:**
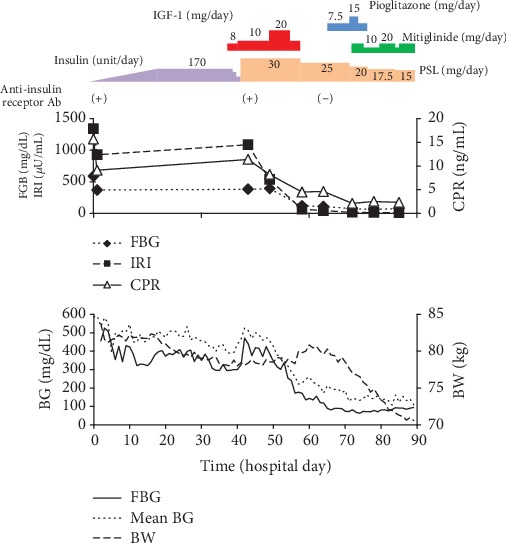
Clinical course of the case. Mean blood glucose was calculated as the average of four measurements (before each meal and sleep (approximately 2 h after dinner)). The upper panel shows an outline of treatment, including the daily insulin dose (unit/day), IGF-1 (mg/day), PSL (mg/day), and oral hypoglycemic agents and changes in the anti-insulin receptor antibody. The middle panel shows the transition of FBG, IRI, and CPR, and the lower panel shows the transition of detailed FBG, mean blood glucose, and body weight. BW: body weight; CPR: C-peptide; FBG: fasting blood glucose; IGF-1: insulin-like growth factor-1; IRI: immunoreactive insulin; PSL: prednisolone; Ab: antibody. The blood glucose level above the upper limit of measurement (600 mg/dL, 33.3 mmol/L) of the self-monitoring blood glucose meter was calculated as 600 mg/dL (33.3 mmol/L).

Figure 2 summarizes the clinical course. The administration of insulin-like growth factor-1 (IGF-1; 0.1 mg/kg = 8 mg/day) was initiated at the hospital on day 37. From the first day of administration, total blood glucose (taken four times per day) decreased by 163 mg/dL (9.06 mmol/L) (Table 3). IGF-1 was gradually increased to 0.125 mg/kg (10 mg/day) on hospital day 40. However, despite this decrease in blood glucose levels in response to IGF-1, blood glucose levels increased transiently following prednisolone administration 4 days after initiating IGF-1 administration. The administration of prednisolone (30 mg/day) was started at hospital day 41, and blood glucose, IRI, and CPR levels decreased sharply within 10 days. Given that prednisolone treatment was effective, IGF-1 administration was terminated on hospital day 59. The insulin receptor antibody was negative in the examination on day 23 after the initiation of prednisolone administration. The dose of prednisolone was gradually decreased, and a maintenance dose of 15 mg/day was set on hospital day 83. On hospital day 64, pioglitazone administration was started; however, FBG levels were sometimes as low as 60–70 mg/dL (3.33–3.89 mmol/L). Thus, pioglitazone was discontinued on hospital day 74. On hospital day 71, mitiglinide (10 mg) was administered prior to breakfast and lunch. Blood glucose levels improved with mitiglinide (20 mg/day) and prednisolone (15 mg/day) treatment, and the patient was discharged. Then, the dose of prednisolone was reduced to 10 mg/day and blood glucose control was good.

Regarding changes in body weight, during the first 40 days, body weight should have been reduced due to an energy-restricted diet, but the weight loss was moderate. The reason was considered to be an anabolic effect by administration of a large amount of insulin. Over the next 25 days, body weight was thought to have increased gradually because of increased body fluid volume due to the mineralocorticoid action of prednisolone. The rapid weight loss of about 10 kg in the last 25 days coincided with the gradual decline in the dosage of prednisolone. This was thought to be due to a decrease in mineralocorticoid action as well as due to the discontinuation of insulin.

There were no serious adverse or unanticipated events observed as well as no change in diet and exercise therapy from start to finish. Food energy intake was restricted to 1600 kcal/day after hospitalization.

In summary, the presence of autoimmune diseases at the time of diagnosis was not obvious in the present case. Acanthosis nigricans was not observed, and extreme or prolonged hypoglycemia was not found. The anti-insulin receptor antibody was positive, and the inhibition rate was 71.3%. Treatment with prednisolone and IGF-1 was effective.

We identified a case of TBIR that developed 6 months after sitagliptin administration, and the patient caught a cold immediately after this development. Examples of relapsed cases of this disease have also been reported, and it is necessary to continue the survey. The possibility of an association between DPP-4 inhibitor and autoimmune disease was reported [16], and it is not possible to rule out the involvement of sitagliptin in the onset of this disease. In addition, we considered that the effect of a viral infection (“cold”) on immunity might have affected TBIR development. Therefore, further investigations are required.

## 3. Results

### 3.1. Study Search

We sent questionnaires to 64 reports. To our knowledge, there were three duplicated cases. There were five cases including two cases in one report (Figure 1). As a result, we approached 66 cases (64–3 + 5) in total and obtained information from 20 cases (separately, we have one case); thus, the recovery rate was 20/66 (30.3%). Overall, the average age of onset of TBIR was 62.3 ± 14.8 (17–84) years. Most patients (90.4%) were ≥50 years, and 66.7% of cases were aged 55–73 years. There were only two cases < 50 years old. There were slightly more males (61.9%) than females. The main patterns of blood glucose levels included 7 cases of hyperglycemia (33.3%), 10 cases of hypoglycemia (47.6%), and 4 cases of both hyperglycemia and hypoglycemia (19.1%). The judgment of the blood glucose pattern (hyperglycemia, hypoglycemia, or both hyperglycemia and hypoglycemia) was made by the doctor in charge. TBIR is a disease in which there is a fluctuation in blood glucose levels; therefore, it is not easy to evaluate it based on uniform criteria. We have determined that the qualitative assessment of glycemic patterns by attending physicians is also important.

The survey items were grouped according to the blood glucose pattern and then compared. Antinuclear antibodies were positive in all seven cases of hyperglycemia. Among the 10 cases of hypoglycemia, 9 cases (90.0%) involved male subjects. Hypoglycemia was observed in 18 cases (85.7%). Among the cases of hypoglycemia, hypoglycemic attack triggered diagnosis in 8 (80.0%) out of 10 cases, and at least 7 (87.5%) out of 8 cases had repeated hypoglycemia. In cases with both hyperglycemia and hypoglycemia, this symptom itself was a trigger for diagnosis in 3 (75.0%) out of 4 cases, and one case was reviewed and diagnosed because IRI was high despite hypoglycemia. In summary, 12 (57.1%) out of 21 cases had hypoglycemia.

Table 4 shows the baseline biochemistry results and characteristics, including sex, age, blood glucose, FIRI, FCPR, and HbA1c. FBG levels were lower than 70 mg/dL (3.89 mmol/L) in 12 cases.

For all items below, the answers obtained for each item were tabulated. Prednisolone was used as treatment in 16 (88.9%) out of 18 cases, at a dose of approximately 30–50 mg (Table 5). The prevalence of TBIR and autoimmune disease was as high as 17 (81.0%) out of 21 cases, but the prevalence of SLE was 8 (38.1%) out of 21 cases, which was a lower rate than previously reported [1]. Other autoimmune diseases were complicated in 13 (65.0%) out of 20 cases (including duplication with SLE) as follows: 5 (23.8%) cases of Sjögren's syndrome, 2 cases of Hashimoto disease, 2 cases of mixed connective tissue disease (MCTD), 1 case of s/o MCTD, 1 case of autoimmune hemolytic anemia, 2 cases of rheumatoid arthritis, 1 case of insulin autoimmune syndrome, and 1 case of polymyalgia rheumatica. There were two cases with Raynaud's symptoms. Antinuclear antibody was positive in 15 (71.4%) out of 21 cases, and 5 (23.8%) out of 21 cases had speckled pattern. On the contrary, 2 (11.1%) out of 18 cases remained double-stranded DNA antibody positive. There were 6 (42.9%) out of 14 cases of *H. pylori* infection, 5 (23.8%) out of 21 cases of acanthosis nigricans, and 3 (15.0%) out of 20 cases of malignant tumors (malignant lymphoma, thymic carcinoma, and gastric cancer). 11 (55.0%) out of 20 cases were anti-insulin antibody positive, and 4 (20.0%) out of 20 cases showed a high binding rate (>70%). The average binding rate of the anti-insulin receptor antibody was 91.4% (71.3%–99.1%) (*n* = 11). Anti-GAD antibodies were recorded in 17 cases and were negative in all cases. The maximum number of insulin units before TBIR diagnosis was 610 units/day. Among the 8 (42.1%) out of 19 cases previously diagnosed with type 2 diabetes, 6 (31.6%) cases involved insulin users. The outcome of TBIR was good. TBIR improved in 14 (93.3%) out of 15 cases. Only one case could not improve and continued to have hypoglycemia in the morning. Diabetes drug use status after TBIR treatment was as follows for 17 cases (with duplicate use): 8 cases had no prescription, 6 cases used *α*-glucosidase inhibitors, 3 cases used glucagon-like peptide-1 receptor agonists, 2 cases used insulin, and 2 cases used biguanide. Some cases also used sodium-glucose cotransporter-2 inhibitors, sulfonylureas, glinides, and thiazolidinediones. Mortality was confirmed in 4 (25.0%) of 16 cases. The other results of the blood and urine tests included low leukocyte count in 8 (40.0%) out of 20 cases, low platelet count in 7 (33.3%) out of 21 cases, enhanced erythrocyte sedimentation rate in 8 (80.0%) out of 10 cases, and overt proteinuria in 9 (45.0%) out of 20 cases.

We divided each item into three groups according to the blood glucose pattern (A, hyperglycemia group; B, hypoglycemia group; and C, both (hyperglycemia and hypoglycemia) group) and compared the groups. Given that the sample sizes of the total sample and each group were modest, nonparametric statistical analyses were performed. For continuous variables, the Kruskal–Wallis test and Steel–Dwass test were performed. For nominal variables, Fisher's exact test was performed, and the Bonferroni and Holm methods were used. There was a significant difference among the three groups for random blood glucose (*P* = 0.009) and significant differences between groups A and B (*P* = 0.038) and B and C (*P* = 0.038). There was also a significant difference among the three groups for sex (*P* = 0.046); however, there was no significant difference between groups B and C (*P* = 0.12, adjusted by the Bonferroni method; *P* = 0.12, adjusted by the Holm method; these were the smallest *P* values in each group comparison). IGF-1 was estimated to be more frequently used by group A than group B (*P* = 0.058). The other items were not significantly different among the three groups.

## 4. Discussion

Insulin resistance syndrome was reported in 1976 by Kahn et al. [17]. Six patients with high insulin resistance and acanthosis nigricans were classified into two types: A and B. Type A is a disease caused by congenital genetic anomalies of the insulin receptor; and type B is an acquired disease caused by the insulin receptor antibody. In addition, type C was also reported. Type C is a disease caused by congenital anomalies in the insulin signal after insulin binds to the insulin receptor [18]. Our study focuses on TBIR.

Although the onset of TBIR is considered more common in women after middle age [1], 61.9% of patients in the present survey were male. In particular, 9 out of 10 patients in the hypoglycemic group were males. It was previously reported that 42.7 years was the average age of Asian patients with TBIR [1]; however, the average age in the present survey was 62.3 ± 14.8 years (17-84 years), which was considered relatively old. There were 7 patients in their 50s, 5 patients in their 60s, and 5 in their 70s, and these patients account for 66.7% of patients aged 55–73 years. Only two cases were <50 years old.

In addition, compared with NIH patients [5], Japanese patients in this study had a higher average age (Japanese 62.3 (17–84) vs. NIH 43.9 (17–64) years), more number of male patients (M/F 13/8 vs. 2/12), and a lower proportion of ANA-positive patients (71.4% vs. 92.9%). However, the proportion of patients with SLE was similar between the two cohorts (38.1% vs. 35.7%). Moreover, compared with previous reports [1, 4] and the report including NIH patients [5], Japanese patients in our study were more likely to have Sjögren's syndrome ([1, 5] = no cases, [4] = 1, and our study = 5). The relatively high incidence of Sjögren's syndrome is considered to be a characteristic of Japanese patients.

The rate at which hypoglycemia triggered the diagnosis was high, and the rate of hypoglycemia was also high overall. It was indicated that hypoglycemia can be seen even in the previous report [1, 2, 19]. In the present survey, hypoglycemia was observed in 85.7% of Japanese cases and was considered a major symptom.

There was a significant difference in the random blood glucose level between the hyperglycemic group (A) and the hypoglycemic group (B) and between the hypoglycemic group (B) and both hyperglycemic and hypoglycemic groups (C) (Table 4). Therefore, we have determined that this grouping has some validity.

16 (88.9%) of 18 cases were treated with prednisolone. Two (11.1%) of 18 cases were treated with immunomodulatory drugs other than prednisolone. While it has been reported that combined immunosuppressive therapy (rituximab, cyclophosphamide, and pulse steroid) is highly effective [5, 20], there were 9 (50.0%) of 18 cases in which TBIR was improved using a combination of immunomodulatory drugs and prednisolone.

### 4.1. IGF-1

IGF-1 may be effective against the increased blood glucose caused by TBIR [2, 6, 12, 19]. Considering that IGF-1 causes insulin-like action via the IGF-1 receptor in skeletal muscle, a reduction in blood glucose was expected in patients suffering from TBIR. IGF-1 was also used in our case and was judged effective because total blood glucose (tested four times/day) decreased by 163 mg/dL (9.06 mmol/L). In this study, five other cases reported the use of IGF-1. The reported effects of IGF-1 on blood glucose were as follows: decrease of 100 mg/dL (5.56 mmol/L) but became ineffective after 1 week, decrease by 30 mg/dL (1.67 mmol/L) after 180 min of administration, FBG decreased by 40 mg/dL (2.22 mmol/L), decrease of up to 150 mg/dL (8.33 mmol/L) of the blood glucose (the total of three measurements before each meal), an average decrease of 30 mg/dL (1.67 mmol/L) with severe blood glucose fluctuation, and no effect. Therefore, even if IGF-1 reduces blood glucose, the decrease is only a maximum of 163 mg/dL (9.06 mmol/L); therefore, it is a limited treatment for marked hyperglycemia.

### 4.2. *H. pylori*

A previous study reported the association between TBIR and *H. pylori* [7]. In the present study, 6 (42.9%) out of 14 cases of infection were reported. For reference, the prevalence of *H. pylori* estimated from the average age (assuming 62.3 years old born in 1948) of Japanese patients with TBIR is 59.9% [21]. In one case, no improvement was observed after eradication. Infection with *H. pylori* causes some immunological abnormalities, which can lead to TBIR [22]. Cases in which no improvement was observed after eradication were complicated by autoimmune hemolytic anemia or chronic thyroiditis possibly because eradication alone could not sufficiently correct the immunological abnormality.

### 4.3. Prognosis

The prognosis of patients suffering from TBIR is poor [4, 5, 9, 19]. On the contrary, the present survey revealed that at least 12 patients were alive and were outpatients at the time of the questionnaire. Only 4 patients were confirmed dead, and 3 of these patients died at 71, 65, and 68 years old, which are considered relatively young in Japan.

### 4.4. DPP-4 Inhibitors

TBIR is an autoimmune disease. Given that DPP-4 inhibitors are therapeutic drugs for diabetes that target a part of the CD26 molecule, which is a costimulatory molecule involved in T cell activation, there is concern over their effect on immune mechanisms. It is presumed that the DPP-4 inhibitor affects the action of CD26, which affects T cells and subsequently B cells, thereby affecting the ability to produce antibodies. In our case, the onset of TBIR was preceded by a common cold and the use of DPP-4 inhibitors. We attempted to investigate the possibility that TBIR was caused by the effects of DPP-4 inhibitors on immunity. In addition to our case, we identified two cases that used DPP-4 inhibitors. It was difficult to find the characteristics. However, there may be an association between DPP-4 inhibitors and the onset of autoimmune diseases, such as bullous pemphigoid and remitting seronegative symmetrical synovitis with pitting edema (RS3PE) syndrome [23–26]. We previously identified and reported a case that developed RS3PE syndrome while using DPP-4 inhibitors. It is possible that DPP-4 inhibitors were also related to TBIR development. However, this is only a hypothesis and will require further consideration via a larger study in the future with the accumulation of many cases.

### 4.5. Gender Differences

It should be noted that men, who were generally at a low risk of autoimmune diseases, showed a higher incidence of hypoglycemia. The relationship between sex hormones and autoimmune diseases has been studied; however, more specifically, the effects of sex hormones on inflammatory cytokines and humoral immunity have also been recognized [27]. Differences in sex hormones may affect the blood glucose pattern and insulin receptor antibody function produced by TBIR. However, this is currently only a hypothesis and will need to be investigated via a larger study in the future.

### 4.6. Strength

In this study, data of some recent Japanese cases were collected. We were able to gather details of the blood glucose pattern, proportion, age, gender, clinical course, inspection results, the state of insulin use before onset, and treatment of each patient. While prednisolone was used the most, the effect of IGF-1 was limited. We have clarified the classification, comparison, and characteristics for each blood glucose pattern. Moreover, we clarified the prevalence of type 2 diabetes before onset, statistical analysis, prognosis, and specific effects of IGF-1. We also noted that the incidence of Sjögren's syndrome in Japanese patients was higher than that in previous reports (non-Japanese). Our study is the first to report these findings. These findings from the present study are valuable as a survey of Japanese cases and can be useful for the development of future treatment.

### 4.7. Limitations

This study has some limitations. With regard to this case study, it was possible that insulin measurement was unstable because of interference of insulin autoantibodies. With regard to this case series, first, the number of cases was limited to 21. Second, not all questions were answered in the questionnaire, and there were missing data, thus possibly reducing the accuracy of the results. Publication bias may also have arisen because only reported cases were targeted. Third, this was a questionnaire survey. There may have been incorrect/inadequate descriptions, or the respondents and nonresponders may have exhibited bias.

## 5. Conclusions

The results of the recent survey showed that male Japanese patients (61.9%) were more affected with TBIR, and there were many hypoglycemic episodes (85.7%). Major treatment was prednisolone (88.9%). Further large-scale investigation is needed to reveal more characteristics of Japanese patients.

## Figures and Tables

**Table 1:** Changes in serum levels of FBG, insulin, C-peptide, HbA1c, glycoalbumin, anti-insulin receptor antibody, and insulin receptor inhibition rate.

Hospital day	0	1	21	43	49	58	64	72	78	85
FBG (mg/dL)	(587) Random	(370) Random	351	382	392	116	109	72	63	76
FBG (mmol/L)	(32.6) Random	(20.6) Random	19.5	21.2	21.8	6.44	6.06	4.00	3.50	4.22
FIRI (*μ*U/mL)	(1340) Random	(929) Random		1088	536	64	43	15	12	11
FCPR (ng/mL)	(15.7) Random	(9.1) Random	5.6	11.4	8.2	4.5	4.6	2.1	2.5	2.3
HOMA-IR						18.3	11.6	2.7	1.9	2.1
HOMA-*β* (%)						435	337	600		305
CPI						3.87	4.22	2.92	3.97	3.03
HbA1c (%)		7.2	10.6	11.9					11.9	
HbA1c (mmol/mol)		55.2	92.3	106					106	
Glycoalbumin (%)		28.1		41.6	46.5	41.3	37.2	32.2	28.1	24.5
Anti-insulin receptor antibody		**+**		**+**			–			
Insulin receptor inhibition rate (%)		71.3		74.7			1.4			

FBG: fasting blood glucose; FIRI: fasting immunoreactive insulin; FCPR: fasting C-peptide; HOMA-IR: homeostasis model assessment for insulin resistance; HOMA-*β*: homeostasis model assessment for beta cell function; CPI: C-peptide index (100x fasting CPR/FBG).

**Table 2:** Laboratory findings.

CBC (D2)		Biochemistry measurements (D2 and D5)		Immunological tests (D8 and D23)	
WBC (/*μ*L)	6800	Na (mEq/L)	133	IgA (mg/dL)	361
Hb (g/dL)	10.9^∗∗^	K (mEq/L)	5.1	IgG (mg/dL)	1068
Plt (×10^3^/mL)	20.3	Cl (mEq/L)	106	IgM (mg/dL)	28^∗∗^
		BUN (mg/dL)	29.7^∗^	C3 (mg/dL)	61^∗∗^
Urinalysis (D1)		Cr (mg/dL)	2.00^∗^	C4 (mg/dL)	20
pH	6.0	TP (g/dL)	6.5	CH50 (U/mL)	49.3^∗^
Blood	+-	Alb (g/dL)	3.3^∗∗^	RF	–
Glucose	4+^∗^	CRP (mg/dL)	0.18	ANA (titer)	–
Protein	2+^∗^	Cortisol (*μ*g/dL)	14.1	Cytoplasmic type	+^∗^
Ketones	–	Free T_4_ (ng/dL)	1.44	ssDNA Ab (AU/mL)	11.0
TP/Cr (mg/g·Cr)	1792.2^∗^	TSH (mU/mL)	0.84	dsDNA Ab (IU/mL)	<5.0
				SS-A (index)	1.1
Diabetes-related measurements (D1–D3 and D10)		Endocrinology measurements (D9 and D10)		SS-B (index)	1.8
FBG (mg/dL)	538^∗^	ACTH (pg/mL)	8.45	Sm Ab (index)	2.1
FBG (mmol/L)	29.9	PRA (ng/mL/h)	1.9	RNP Ab (index)	5
Fasting IRI (*μ*U/mL)	Impossible to measure^#^	ALDS (pg/mL)	81	MPO-ANCA (IU/mL)	0.5
Fasting CPR (ng/mL)	10.3^∗^	CS (*μ*g/dL)	14.1	PR3-ANCA (IU/mL)	0.5
Proinsulin (pmol/L) (D10)	942^∗^	GH (ng/mL)	0.842		
HbA1c (%)	7.2	SM-C (ng/mL)	48		
HbA1c (mmol/mol)	55.2	U-metanephrine (mg/L)	0.02^##^		
Glycoalbumin (%)	28.1^∗^	U-normetanephrine (mg/L)	0.11^##^		
Anti-GAD Ab (U/mL)	<0.3	U-cortisol (*μ*g/L)	13.0^##^		
Anti-insulin Ab (%)	7.4^∗^				
IA-2 Ab (U/mL)	0.8^∗^				
Anti-insulin receptor Ab (%)	71.3^∗^				

Ab: antibody; ACTH: adrenocorticotropic hormone; ALDS: aldosterone; ANA: antinuclear antibody; BG: blood glucose; CPR: C-peptide; CRP: C-reactive protein; CS: cortisol; dsDNA Ab: double-stranded DNA antibodies; GAD: glutamic acid decarboxylase; Hb: hemoglobin; IA-2: islet antigen 2; IRI: immunoreactive insulin; PRA: plasma renin activity; GH: growth hormone; MPO: myeloperoxidase; Plt: platelet; PR3: proteinase-3; RF: rheumatoid factor; RNP Ab: ribonucleoprotein antibodies; ssDNA Ab: single-stranded DNA antibodies; Sm Ab: Smith antibodies; SM-C: somatomedin C; TP/Cr: total protein/creatinine; TSH: thyroid-stimulating hormone; WBC: white blood cell. ^∗^Above the reference range; ^∗∗^below the reference range; ^#^it was considered impossible to measure due to the extremely high value and the influence of insulin antibody; ^##^because the urine collection test was not allowed at our hospital at that time, these items were evaluated using early morning urine.

**Table 3:** Changes in mean BG, total BG, insulin dose, and PSL dose before and after starting IGF-1.

Hospital day	IGF-1 dose (mg/kg)	Insulin dose (unit/day)	PSL (mg/day)	Mean BG (mg/dL)/(mmol/L)	Total BG (mg/dL)/(mmol/L)	Difference of BG (mg/dL)/(mmol/L)
35	—	170	—	435/24.2	1738/96.6	
36	—	170	—	435/24.2	1738/96.6	163/9.06
37	0.1	170	—	394/21.9	1575/87.5	
38	0.1	140	—	389/21.6	1556/86.4	
39	0.1	100	—	395/21.9	1578/87.7	
40	0.125	50	—	438/24.3	1751/97.3	
41	0.125	0	30	466/25.9	1863/103.5	
42	0.125	0	30	526/29.2	2103/116.8	
43	0.125	0	30	511/28.4	2045/113.6	

Mean blood glucose was calculated as the average of four measurements (before each meal and sleep (approximately 2 h after dinner)). Total blood glucose was calculated as the total amount of the above four measurements. IGF-1: insulin-like growth factor-1; PSL: prednisolone; BG: blood glucose.

**Table 4:** Clinical characteristics and biochemistry measurements at baseline.

Group	A	B	C			
Blood glucose pattern	Hyperglycemia	Hypoglycemia	Hyperglycemia and hypoglycemia	Total	*P* value (Kruskal–Wallis test)	*P* value (Steel–Dwass test)
*n*	7	10	4	21		
Sex (M/F)	3/4	9/1	1/3	13/8	*P* = 0.046	*P* > 0.05
Age (years)	60.1 ± 9.2	63.6 ± 19.8	63.0 ± 10.1	62.3 ± 14.8	*P* > 0.05	—
FBG (mg/dL)	180 ± 178	54 ± 18	91 ± 79	102 ± 117	*P* > 0.05	—
FBG (mmol/L)	9.98 ± 9.89	2.98 ± 1.01	5.06 ± 4.39	5.67 ± 6.48		
Random BG (mg/dL)	460 ± 129	128 ± 84	327 ± 105	291 ± 174	*P* = 0.009	*P* = 0.038 (A-B), *P* = 0.038 (B-C)
Random BG (mmol/L)	25.6 ± 7.15	7.10 ± 4.66	18.1 ± 5.82	16.2 ± 9.66		
FIRI (mU/L)	542 ± 317	451 ± 774	492 ± 177	490 ± 563	*P* > 0.05	—
FCPR (ng/mL)	5.2 ± 4.3	9.0 ± 9.5	4.1 ± 2.9	6.9 ± 7.3	*P* > 0.05	—
HbA1c (%)	9.8 ± 2.6	7.6 ± 1.7	8.0 ± 2.6	8.4 ± 2.4	*P* > 0.05	—
HbA1c (mmol/mol)	83.3 ± 28.1	59.2 ± 19.1	52.6 ± 22.1	67.0 ± 25.4		

BG: blood glucose; FBG: fasting blood glucose; FIRI: fasting immunoreactive insulin; FCPR: fasting C-peptide. For all items, the answers obtained for each item were tabulated.

**Table 5:** Treatment and comorbidity.

Group	A	B	C		
Blood glucose pattern	Hyperglycemia	Hypoglycemia	Hyperglycemia and hypoglycemia	Total	*P* value (Fisher's exact test)
*n*	7	10	4	21	
PSL administration (%)	85.7	100.0	75.0	88.9	*P* > 0.05
Immunosuppressant administration other than PSL (%)	42.9	57.1	100.0	61.1	*P* > 0.05
IGF-1 administration (%)	71.4	14.3	0	37.5	*P* > 0.05
SLE (%)	42.9	40.0	25.0	38.1	*P* > 0.05
Autoimmune diseases other than SLE (%)	57.1	66.6	75.0	65.0	*P* > 0.05
*Helicobacter pylori* positive (%)	66.7	37.5	33.3	42.9	*P* > 0.05
Diagnosis of T2DM before the onset of TBIR (%)	28.6	44.4	66.7	42.1	*P* > 0.05
ANA positive (%)	100.0	60.0	50.0	71.4	*P* > 0.05

Each item other than the number of patients is shown in the corresponding ratio. ANA: antinuclear antibody; BG: blood glucose; IGF-1: insulin-like growth factor-1; PSL: prednisolone; SLE: systemic lupus erythematosus; T2DM: type 2 diabetes mellitus; TBIR: type B insulin resistance. For all items, the answers obtained for each item were tabulated.

## Data Availability

The data that support the findings of this study are available from the corresponding author on reasonable request.
